# ^18^F-FDG-PET Scanning Confirmed Infected Intracardiac Device-Leads with* Abiotrophia defectiva*

**DOI:** 10.1155/2016/6283581

**Published:** 2016-03-27

**Authors:** Sonja van Roeden, Hans Hartog, Vivian Bongers, Steven Thijsen, Sanjay Sankatsing

**Affiliations:** ^1^Department of Internal Medicine, Diakonessenhuis, 3582 KE Utrecht, Netherlands; ^2^Department of Cardiology, Diakonessenhuis, 3582 KE Utrecht, Netherlands; ^3^Department of Nuclear Medicine, Diakonessenhuis, 3582 KE Utrecht, Netherlands; ^4^Department of Microbiology, Diakonessenhuis, 3582 KE Utrecht, Netherlands

## Abstract

*Abiotrophia* species are relatively slow growing pathogens, which may be present as commensal flora. However, invasive infections are frequently reported, like endocarditis, septic arthritis, osteomyelitis, and many other types of infection. In this case report we describe a 65-year-old male patient with an intracardiac device- (ICD-) lead infection caused by* Abiotrophia defectiva*. Diagnosis was confirmed by ^18^F-FDG-PET scanning. This is remarkable, since* Abiotrophia defectiva* is a slow growing pathogen causing low-grade infections. This case demonstrates that although infection of ICD-leads cannot be excluded in case of ^18^F-FDG-PET-negative findings, positive findings are highly suggestive for infection.

## 1. Introduction

*Abiotrophia* species* are nutritionally variant streptococci (NVS)*, with high similarity to* Granulicatella* spp.* Abiotrophia defectiva* is the only species in the genus. It may be difficult to distinguish subtypes of* Abiotrophia* and* Granulicatella* spp. by standard biochemical tests for clinical microbiological laboratories; therefore they are frequently reported as* Abiotrophia* or* Granulicatella* species. It is a relatively slow growing pathogen compared to other streptococci; therefore cultivation and identification can be challenging [1].

*Abiotrophia* species may be present as commensal flora of the oral cavity and the urogenital and gastrointestinal tracts [1]. Invasive infections are frequently reported in literature, for example, continuous ambulatory peritoneal dialysis-associated peritonitis, endocarditis, endovascular graft infection, osteomyelitis, pyogenic discitis, septic arthritis, spontaneous brain abscess, and ocular infections [1–5]. In this case report, we present a rare infection caused by* Abiotrophia* species, namely, a patient with ^18^F-FDG-PET scanning that confirmed infected ICD-leads with* Abiotrophia* species.

## 2. Case Report

**Figure 1:**
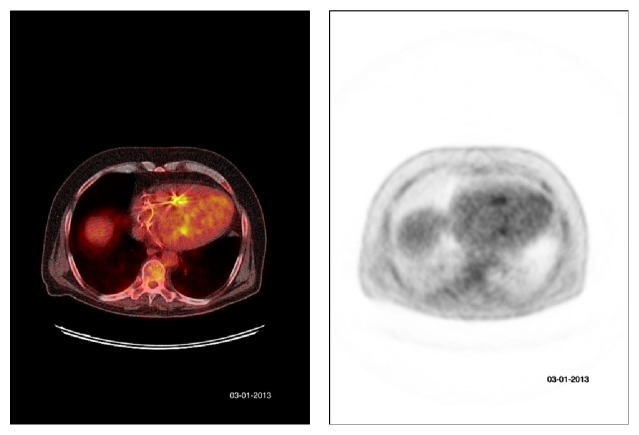
^18^F-FDG-PET scanning demonstrates pathologic activity by the tip of both ICD-leads.

A 65-year-old male was referred to the outpatient clinic Internal Medicine in February 2013 with complaints of pain and stiffness of his neck since three months. He reported two episodes of chills without fever. He had no weight loss, night sweats, traumatic injury, pain, or stiffness elsewhere in his body. His medical history revealed systolic and diastolic heart failure with a gated pool ejection fraction of 26%, for which he received cardiac resynchronization therapy with an intracardiac device (CRTD) (type: Sorin Paradym 8750) in July 2010. Physical examination revealed no abnormalities, explicitly no fever, cardiac murmurs, or skin abnormalities. Laboratory investigations showed a raised C-reactive protein of 68 mg/L (normal range: <10 mg/L), erythrocyte sedimentation rate >95 mm/H (normal range: <15 mm/hour), and leukocytes of 9.3 × 10^9^/L (normal range: <10 × 10^9^/L). A spondylodiscitis was considered; however there was no evidence for spondylodiscitis on a CT-scan of the cervical spine or bone scintigraphy. Blood cultures were taken and 10 out of 14 turned positive for* Abiotrophia defectiva*, confirmed with mass spectrometry (MALDI-TOF). Treatment with high dose intravenous penicillin (12 million units/24 hrs) was started. An intravascular focus, for example, endocarditis, was suspected. Duke's criteria for infective endocarditis were not met; transesophageal echography of the heart revealed no vegetations on the cardiac valves or leads of the ICD. ^18^F-FDG-PET scanning demonstrated pathologic activity by the tip of both ICD-leads and at a right palatal tonsil (Figure 1). Removal of the ICD-leads was impossible due to extensive fibrosis caused by the infection. Treatment with high dose intravenous penicillin was continued for six weeks and the patient was additionally treated with clindamycin (600 milligrams three times/24 hrs) orally for six weeks. The ear-nose-throat specialist found no abnormalities on physical examination. The initial neck complaints disappeared after physical therapy. The infected ICD-leads could be the result of bacteriemia due to infection elsewhere; a potential dental focus was found. Two months after discontinuation of antibiotic therapy, laboratory findings did not indicate infection, and repeated ^18^F-FDG-PET scanning three months later showed no pathological activity: neither at the tips of the ICD-leads, nor elsewhere in the body.

## 3. Discussion

To our knowledge, only one suspected case of infected ICD and/or ICD-leads by* Granulicatella* spp. is reported in literature, with no confirmation by ^18^F-FDG-PET scanning nor removal of the ICD [4]. Infected ICDs coincide with a high mortality, up to 17% [6]. Infection may occur even >20 years after implantation and may be challenging to diagnose [7]. In case of signs and symptoms of infection, for example, fever, local symptoms, or positive blood cultures, echocardiography may indicate infection of intracardiac located parts of an ICD. However, infection of the extracardiac portion may be overlooked [8]. Vegetations on leads or ICDs can be suspected on CT-scan, but these findings are subtle and nonspecific and may represent thrombus or artifacts caused by metal parts. It is suggested that ^18^F-FDG-PET scanning is a promising and helpful tool in diagnosing an infected ICD and ICD-leads. Multiple patients with ^18^F-FDG-PET positive infected devices or leads caused by other species are reported with a sensitivity varying from 24 to 100% and a specificity varying from 79 to 100% [9, 10]. However, diagnosing infected ICD-leads by ^18^F-FDG-PET scanning is not free from pitfalls [9]. False positivity may be caused by mechanical rubbing of devices against thoracic muscles, leading to mild inflammation. Secondly, the size of the vegetations on the leads may be too small for detection by ^18^F-FDG-PET scanning, potentially causing false negative results [9]. However, specificity is high and, in case of pathological uptake around leads, infection is very likely [9].

In conclusion, we report the first ^18^F-FDG-PET positive intracardiac device lead infection caused by* Abiotrophia defectiva*, demonstrating that ^18^F-FDG-PET is a useful, noninvasive tool for detection and follow-up of infected ICD-leads. Although infection of ICD-leads cannot be excluded in case of ^18^F-FDG-PET-negative findings, positive findings are highly suggestive for infection. Even though* Abiotrophia defectiva* grows relatively slow, ^18^F-FDG-PET findings were positive. This demonstrates that ^18^F-FDG-PET may also be helpful for diagnosing low-grade infections of ICD-leads.
